# Sonographic Visualization of a Tortuous Optic Nerve: Case Report of a Novel Finding on Point-of-Care Ultrasound

**DOI:** 10.5811/cpcem.43504

**Published:** 2025-08-24

**Authors:** Lucas Delicio, Adam Pearl, Vu Huy Tran

**Affiliations:** HCA Aventura Hospital, Emergency Department, Aventura, Florida

**Keywords:** *idiopathic intracranial hypertension*, *point-of-care ultrasound*, *optic nerve tortuosity*, *magnetic resonance imaging*

## Abstract

**Introduction:**

Idiopathic intracranial hypertension is a disorder typically affecting females with common complaints of headaches and visual disturbances. Diagnostic criteria have been described with clinical findings, high opening pressures in lumbar punctures, and magnetic resonance imaging (MRI) findings.

**Case Report:**

A 36-year-old female presented with double vision and headaches. Point-of-care ultrasound demonstrated tortuosity of the optic nerve, a finding previously described in MRI studies, which may serve as an additional marker for ideopathic intracranial hypertension.

**Conclusion:**

This case highlights the potential of point-of-care ultrasound to detect tortuous optic nerves, which may help in the early diagnosis of ideopathic intracranial hypertension, facilitating more timely and effective management.

## INTRODUCTION

Idiopathic intracranial hypertension (IIH), or pseudotumor cerebri, primarily affects females 15–44 years of age and is characterized by increased intracranial pressure without identifiable structural causes.^1^ Symptoms include headaches, visual disturbances and, occasionally, double vision.^1^ While IIH diagnosis typically relies on clinical criteria, point-of-care ultrasound (POCUS) is emerging as a valuable bedside diagnostic tool in detecting increased intracranial pressure. This case introduces the novel finding of a tortuous optic nerve on POCUS, which could serve as an additional marker for IIH.

## CASE REPORT

A 36-year-old female with a medical history of Chiari malformation, iron deficiency, and uterine fibroids presented to the emergency department (ED) with complaints of headaches, double vision, and elevated blood pressure for the prior two days. The patient also noted intermittent episodes of dizziness over the preceding three weeks. On physical examination, the patient exhibited normal cranial nerve function, intact motor strength, and absence of nystagmus. The reflexes were 2+, and her gait was normal. The remainder of the exam was unremarkable. Laboratory results were non-contributory, and computed tomography imaging of the brain, along with venography, revealed tonsillar ectopy.

**Image 1:**
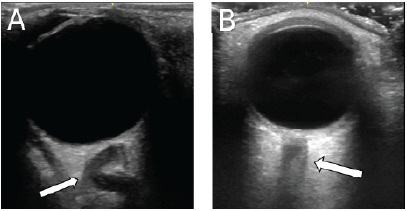
A) Point-of-care ultrasound image demonstrating tortuous optic nerve of the left eye from the patient (arrow), and B) a normal optic nerve of the left eye unrelated to the patient (arrow).

Ocular POCUS was performed, revealing a dilated optic nerve sheath bilaterally with elevation of the left optic disk, consistent with papilledema. Notably, the left optic nerve exhibited a tortuous, rather than linear, appearance (Image 1). Based on these findings, the patient was admitted for further evaluation by neurology to exclude IIH.

### Hospital Course

**Image 2:**
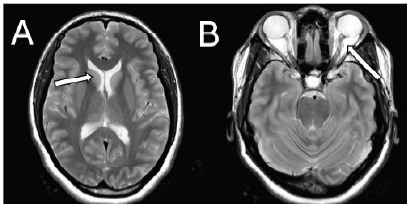
Magnetic resonance imaging demonstrating slit ventricles (white arrow in A) and tortuous optic nerve (white arrow in B).

The patient underwent magnetic resonance imaging (MRI), which showed slit-like ventricles and tortuosity of the left optic nerve (Image 2). These findings, along with the clinical presentation, supported the diagnosis of IIH. The patient was started on acetazolamide and discharged with close neurology follow-up.

## DISCUSSION

Idiopathic intracranial hypertension, also known as pseudotumor cerebri, is a rare but well-known condition typically affecting women 15–44 years of age, with a strong association to obesity.^1^ Symptoms can include blurred vision, neck pain, dizziness, double vision, headaches, and pulsatile tinnitus. The pathophysiology of IIH is still unknown, as it is not due to fluid buildup like other common causes of increased intracranial pressure (eg, hemorrhage). Although the precise pathophysiology remains unclear, proposed mechanisms of IIH include CSF flow diversion and venous sinus stenting.^2^ A diagnosis is typically made after exclusions of other causes of intracranial hypertension are ruled out. The Modified Dandy Criteria are frequently used to aid in diagnosis, incorporating symptoms of increased ICP, no localizing neurological findings, lumbar puncture opening pressure of greater than 25 centimeters of water with negative CSF analysis, absence of abnormalities with the ventricular system, and no discernable etiology for increased ICP.^3^ During evaluation, imaging is commonly obtained to rule out secondary causes of increased ICP, with MRI findings often aiding in characterization. Over the last few decades MRI findings have been shown to increase specification of IIH with signs including empty turcica, venous sinus stenosis, posterior globe flattening, optic nerve sheath dilation, and slit-like ventricles.^4^

*CPC-EM Capsule*

What do we already know about this clinical entity?

*Idiopathic intracranial hypertension presents with well described symptoms and exam findings previously described in magnetic resononance imaging studies*.

What makes this presentation of disease reportable?

*A point-of-care ultrasound demonstrated a tortuous optic nerve, expanding ultrasound’s role in identifying subtle neurological findings*.

What is the major learning point?

*Ultrasound can potentially identify tortuous nerves, highlighting its potential as a quick, accessible diagnostic tool in the emergency department*.

How might this improve emergency medicine practice?

*Ultrasound could aid in identifying subtle neurological findings at bedside*.

A systematic review by Kwee et al highlighted optic nerve tortuosity on MRI as having low sensitivity (36.9%) but high specificity (88.4%) for diagnosing IIH across seven pooled studies.^5^–^6^ Although not commonly detected, this feature can be useful in cases where other diagnostic signs are inconclusive; it has been used to aid in diagnosis of patients who have common symptoms associated with IIH.^5^–^6^ Our patient’s MRI demonstrated nearly all findings consistent with her end diagnosis, IIH. Unique to our case was the use of POCUS to discern a torturous optic nerve before the MRI imaging was done, which raised our suspicion for IIH and necessary neurology evaluation.

Increased optic nerve sheath diameter and optic disc elevation seen on POCUS has been well described as a finding associated with IIH.^7^ Optic nerve elevation refers to a hyperechoic prominence that extends into the vitreous. The height of the prominence has been shown to correlate with severity of the increased intracranial pressure. Optic nerve elevation measurement cutoff has been proposed to be 0.3–1 millimeter (mm), with a sensitivity of 70–90% and specificity of 69–100%.^8^ The optic nerve sheath diameter has been studied and shown to correlate with increased intracranial pressure. An optic nerve sheath diameter greater than 5.6 mm was found to have a sensitivity of 93.75% and a specificity of 86.67% for increased intracranial pressure in Korean adults.^9^ This proves to be useful in a setting where early diagnosis of increased intracranial pressure improves patient outcomes.

The torturous optic nerve route on POCUS has not been previously well established in the literature. Given our patient’s MRI findings and similar findings on POCUS, we believe that this finding may aid in the diagnosis of increased intracranial pressure and possibly IIH. It Is important to note that other conditions may cause a tortuous optic nerve, such as neurofibromatosis, connective tissue disease, optic gliomas, and glaucoma.^10^–^11^ In the ED, POCUS can be a valuable tool for primary screening. The presence of a tortuous optic nerve on ultrasound may serve as a useful diagnostic clue, raising suspicion for increased intracranial pressure and potentially indicating conditions such as IIH.

## CONCLUSION

This case underscores the potential of point-of-care ultrasound in detecting tortuous optic nerves, which may serve as a novel marker for increased intracranial pressure and idiopathic intracranial hypertension. Further research is needed to validate this finding and establish it as a reliable diagnostic clue in clinical practice. In the meantime, POCUS remains a promising tool for early diagnosis, potentially leading to better outcomes in patients with idiopathic intracranial hypertension.
